# Scale-up of a chronic care model-based programme for type 2 diabetes in Belgium: a mixed-methods study

**DOI:** 10.1186/s12913-023-09115-1

**Published:** 2023-02-09

**Authors:** Katrien Danhieux, Veerle Buffel, Roy Remmen, Edwin Wouters, Josefien van Olmen

**Affiliations:** 1 Family Medicine and Population Health, University of Antwerp, Antwerp, Belgium; 2 Institute of Tropical Medicine Antwerp, Antwerp, Belgium; 3 Center for Population, Family and Health, University of Antwerp, Antwerp, Belgium

**Keywords:** Type 2 diabetes, Chronic care, Primary care, Chronic care model, Primary care nurse, Scale-up, Integrated care

## Abstract

**Background:**

Type 2 diabetes (T2D) is an increasingly dominant disease. Interventions are more effective when carried out by a prepared and proactive team within an organised system — the integrated care (IC) model. The Chronic Care Model (CCM) provides guidance for its implementation, but scale-up of IC is challenging, and this hampers outcomes for T2D care. In this paper, we used the CCM to investigate the current implementation of IC in primary care in Flanders (Belgium) and its variability in different practice types.

**Methods:**

Belgium contains three different primary-care practice types: monodisciplinary fee-for-service practices, multidisciplinary fee-for-service practices and multidisciplinary capitation-based practices. Disproportional sampling was used to select a maximum of 10 practices for each type in three Flemish regions, leading to a total of 66 practices. The study employed a mixed methods design whereby the Assessment of Chronic Illness Care (ACIC) was complemented with interviews with general practitioners, nurses and dieticians linked to the 66 practices.

**Results:**

The ACIC scores of the fee-for-service practices — containing 97% of Belgian patients — only corresponded to basic support for chronic illness care for T2D. Multidisciplinary and capitation-based practices scored considerably higher than traditional monodisciplinary fee-for-service practices. The region had no significant impact on the ACIC scores. Having a nurse, being a capitation practice and having a secretary had a significant effect in the regression analysis, which explained 75% of the variance in ACIC scores. Better-performing practices were successful due to clear role-defining, task delegation to the nurse, coordination, structured use of the electronic medical record, planning of consultations and integration of self-management support, and behaviour-change intervention (internally or using community initiatives). The longer nurses work in primary care practices, the higher the chance that they perform more advanced tasks.

**Conclusions:**

Besides the presence of a nurse or secretary, also working multidisciplinary under one roof and a capitation-based financing system are important features of a system wherein IC for T2D can be scaled-up successfully. Belgian policymakers should rethink the role of paramedics in primary care and make the financing system more integrated. As the scale-up of the IC varied highly in different contexts, uniform roll-out across a health system containing multiple types of practices may not be successful.

**Supplementary Information:**

The online version contains supplementary material available at 10.1186/s12913-023-09115-1.

## Background

Type 2 diabetes (T2D) is on the rise throughout the world. If current trends continue, 700 million adults will have T2D by 2045, affecting 11% of the world’s population. Obviously, the economic impact of the illness is also expected to grow [1], but can be largely mitigated by preventing complications [2]. Although consensus exists about the ideal diabetes management strategy [3] — encompassing lifestyle treatment, administering medication and basic check-ups such as blood tests, which are all technically easy — the level of implementation stays low [4, 5]. As T2D is a chronic disease, which is often not marked by symptoms in the first phase, it requires a proactive approach, different to the approach used for acute diseases. The need for attention to planning, patient education and empowerment results in more complex and difficult disease management. Integrated care (IC), which is a commonly known concept that many organisations strive for [6], wants to overcome these barriers and the Chronic Care Model (CCM) [7] aims to provide the building blocks to shape it in order to improve the quality of care for all patients.

The CCM approach has been proven effective in improving quality of care in some studies (although not overwhelming) [8, 9], and also in the field of T2D care [10, 11]. However, the success of the implementation of the CCM for T2D care can be very variable and context-specific [12–16]. Examples of such contextual factors are financing structures [17, 18], organisation culture and human resources [19]. When we want successful interventions to impact the population’s health, they need to be spread and scaled-up. The study of scale-up is still in its infancy, especially in primary care [20]. It is clear that in any case, scale-up is difficult, sometimes unsuccessful and the effects of the intervention often attenuate [21–25]. Specifically, there have been no studies that address the variability of the success of scale-up of chronic care programmes in primary care. What happens after the pilot project, when the programme is put into policy, but researchers and intensive support have left? In which contexts can these programmes be scaled-up successfully and why?

In this study, we investigated the current implementation of IC for T2D in primary care in Flanders (Belgium), by using the CCM. The aim was to study the variability of the implementation of IC for T2D across different types of primary care practices and to examine which elements in their practice organisation explain this variability and why. This was done using both quantitative and qualitative methods. In doing so, we will be able to better understand what circumstances are needed to implement IC. As such, our findings will better inform policymakers and researchers about the real current state of IC and their barriers and levellers to implementation, so they can target their efforts more efficiently.

## Methods

### Research context

Belgium has a healthcare system based on compulsory health insurance, which covers 99% of the population for a broad range of services. Free choice of provider is an important principle and there is no gatekeeping function, so patients can visit multiple general practitioners (GPs) and have direct access to specialist care. However, financial incentives, such as the Global Medical Record (patients who opt in for the global medical record allow a GP practice to manage their medical information and will have lower co-payments) are used to channel patient behaviour according to the gatekeeper model.

In primary care, GPs can choose between two payment models: fee-for-service or capitation. Most GPs (94%) opt for the fee-for-service model and work as independent providers — the services they provide are reimbursed by health insurance. In 2018, 61% of them worked solo and the other 39% in group practices with other GPs [26]. Only 6% of the GPs worked within the capitation system. In these practices, patients have to register to the practice, and the practice receives a monthly fee based on the population they care for. Patients do not pay user charges and cannot consult other GPs.

Contrary to other countries, nurses rarely support GPs in the classic fee-for-service practices in Belgium, as there is no remuneration system. Recently, some innovative GPs have involved nurses (whom they pay from their own revenues) in their practice. On the other hand, all of the capitation practices also offer care by nurses and many also have other providers, such as dieticians, physiotherapists or psychologists. Dieticians are certified healthcare providers and have a role in the treatment of diabetic patients. The reimbursement of their services is, however, limited to two consultations a year for these patients. Sometimes, in fee-for-service practices, GPs ask dieticians to do consultations within their practice.

Since 2009, a care pathway for patients with T2D has been established, in order to organise cooperation between patients, their GPs and endocrinologists. If patients register to this system they get reimbursement for self-management material and consultations with a diabetes educator, who is a nurse or dietician with a special accreditation.

### Study design

A mixed method design was used to answer both research questions: which primary care practices have implemented IC better (quantitative) and why is this the case (qualitative)? The Assessment of Chronic Illness Care (ACIC) is a tool developed to assess levels of care based upon the six elements of the CCM: Health Organisation, Community Linkages, Self-management Support, Decision Support, Delivery System Design and Clinical Information Systems (see Table 1) [27]. It was used before in a Belgian study concerning T2D care [28], translated to Dutch [29], and in many other international studies, it has proven to be a validated instrument [11, 30–34]. Based on the tool, both quantitative data (as a score between 0 and 11) and qualitative data (as a description of how the practice implements each element) are collected. The quantitative scores should be interpreted as follows: between 0 and 2 = limited support for chronic illness care (CIC), between 3 and 5 = basic support for CIC, between 6 and 8 = reasonably good support for CIC, between 9 and 11 = fully developed CIC. Element 1 (Health Organisation) and question 2.3 (Regional Health Plans) concern questions about care on a regional level and, therefore, stakeholders on this level were interviewed. The other questions were answered through primary care practices, as these are questions at the operational level. We used the ACIC tool to collect both quantitative and qualitative data; therefore, this design of this study can be defined as a fully mixed concurrent equal status design [35].

**Table 1:** Elements of the Chronic Care Model

Health Organisation	Chronic illness management programmes can be more effective if the overall system (organisation) in which care is provided is oriented and led in a manner that allows for a focus on chronic illness care
Community Linkages	Linkages between the health system and community resources play important roles in chronic illness management
Self-management Support	Effective self-management support can help patients and families cope with the challenges of living with and treating chronic illnesses and reduce complications and symptoms
Decision Support	Effective chronic illness management programmes assure that providers have access to evidence-based information necessary to care for patients—decision support. This includes evidence-based practice guidelines or protocols, specialty consultation, provider education and activating patients to make provider teams aware of effective therapies
Delivery System Design	Evidence suggests that effective chronic illness management involves more than simply adding additional interventions to a current system focused on acute care. It may necessitate changes to the organisation of practice that impact provision of care
Clinical Information Systems	Timely, useful information about individual patients and populations of patients with chronic conditions is a critical feature of effective programmes, especially those that employ population-based approaches

This study is part of the Scale-Up diabetes and hYpertension care (SCUBY) project, which aims to scale-up IC through the development and evaluation of roadmap strategies in different types of healthcare systems in Belgium, Cambodia and Slovenia [36, 37].

### Sampling

Primary care practices in Belgium can be categorised based on two dimensions: (a) provider-payment mechanism: fee-for-service or capitation and (b) whether the practice was multidisciplinary or not. Being a multidisciplinary practice was defined as having a nurse or dietician providing services under the same roof as the GP, as these two are the main paramedics (non-physicians) in the treatment of T2D. Based on these two dimensions, four types of practices can be defined, theoretically. However, in reality, monodisciplinary capitation practices do not exist. Therefore, three different practice types were sampled: monodisciplinary fee-for-service practices, multidisciplinary fee-for-service practices and multidisciplinary capitation practices.

In order to reach enough variation, three regions in which a big spread of all of these practices was present were chosen: the urban regions, Antwerp and Ghent and the semi-rural region, the Campine. Disproportional sampling was used by selecting a maximum of ten practices for each type in every region. Within the monodisciplinary practices, an equilibrium of solo and group practices was strived for. In every region, one stakeholder in charge of the implementation of chronic care at the local level was interviewed.

### Data collection

An interview guide, based on the ACIC was developed. Open-ended questions were used to ask the practitioners to describe their practice. Where needed, topics were explored deeper and the practitioners were asked for an explanation. This methodology was used to prevent the survey from being a checklist in which the respondents could easily answer affirmatively, although that may not be the case. In two different pilot visits, the interview guide was tested and refined.

Based on publicly-available lists, primary care practices were categorised and randomised. We reached out to the practices by e-mail and phone to ask for their participation until the maximum of 10 practices was reached, or all were asked, for each type of practice, in each region. In each practice, two researchers interviewed the GP, nurse and dietician (if available) separately. One researcher was the main interviewer, the other made notes and asked additional questions. After each visit a field note was written by the researchers, summarising their findings. Subsequently, both researchers filled in the ACIC independently and discussed their scores, after which a consensus score was defined. The scores obtained after the interviews with the regional stakeholder counted for all the practices in that particular region. For every element, the mean was calculated and the total score was calculated as the final mean of these individual means. Due to the lockdown following the COVID-19 pandemic, the last few interviews had to be performed by video call. Practitioners were asked to answer the questions based on their experiences before the pandemic.

### Analysis

The quantitative part consists of two steps. First, bivariate analyses were performed to look for significant differences in the ACIC scores among the different types of practices and regions, using ANOVA tests and post hoc Bonferroni tests. Second, multivariate regression analyses were performed to estimate the relation between various GP-practice characteristics and the ACIC scores, while controlling for region. We relied on a stepwise procedure: in model 1 the variable ‘financing type’ was used; in model 2, the variables ‘a dietician’ and ‘a nurse’ were added; and in model 3, the variable ‘a secretary’ was added. All models were controlled for ‘region’. Statistical analyses were performed using SPSS Statistics.

The field notes of the researchers were used for the qualitative analysis. These notes provided information on the different mechanisms that providers used to organise their practice. In this way, the differences between the different practices were explained. Qualitative analysis was performed with NVivo software.

## Results

A total of 66 practices participated and an interview with one member of all disciplines present (GPs, nurses, dieticians) was conducted. The response rate differed across subgroups: 16% among monodisciplinary practices, 49% among multidisciplinary fee-for-service practices and 94% among multidisciplinary capitation practices. Table 2 shows participating practices’ characteristics. We will first outline the quantitative data and then explain our results qualitatively, by each element of the CCM.

**Table 2:** Practice characteristics (*n* = 66)

Variable	Value	N	Percent
**Region**	Antwerp	26	39%
	Ghent	23	35%
	Campine	17	26%
**Type of GP practice**	Monodisciplinary, fee-for-service, solo	16	24%
	Monodisciplinary, fee-for-service, group	14	21%
	Multidisciplinary, fee-for-service	19	29%
	Multidisciplinary, capitation	17	26%
**Nurse**	Yes	27	41%
	No	39	59%
**Dietician**	Yes	24	36%
	No	43	65%
**Secretary**	Yes	45	68%
	No	21	32%
**Age of interviewed GP (YEARS)**	< 35	29	44%
	35–50	16	24%
	> 50	21	32%
**Gender of interviewed GP**	Male	33	50%
	Female	33	50%

### ACIC scores by region

There was no significant difference between the regions on the total ACIC score and its separate elements. All elements, except the first two, were measured on the practice level, so *p*-values could be calculated (Table 3). The first two elements were scored using the answers of the meso-level stakeholders (so that there was no variation between the practices in one region, resulting in the statistical analysis not making sense). The practices in the region of Ghent scored the highest, followed by the regions of the Campine and Antwerp, but all in the range of basic support for CIC.

**Table 3:** Average ACIC scores by region

ACIC elements	Urban				Rural		
	Ghent		Antwerp		Campine		
	x̄	sd	x̄	sd	x̄	sd	*p*-value*
1. Health Organisation	8.17	N/A	4.67	N/A	5.83	N/A	N/A
2. Community linkages	3.84	N/A	2.53	N/A	4.24	N/A	N/A
3.a Self-management support	5.22	1.99	4.19	1.82	4.18	1.60	0.10
3.b Decision support	3.63	1.42	3.62	1.09	3.97	0.97	0.58
3.c Delivery system design	4.35	2.64	3.74	1.66	4.26	2.11	0.57
3.d Clinical information system	4.38	2.73	4.42	2.16	3.50	1.82	0.39
**Total ACIC score**	**4.93**	**1.64**	**3.86**	**1.26**	**4.33**	**1.10**	**N/A**

^*^ANOVA test; *sd* Standard deviation, *N/A* Not applicable

### ACIC scores by type of GP practice

Capitation practices scored significantly higher than multidisciplinary fee-for-service practices, which scored, on their turn, higher than monodisciplinary practices on all the ACIC elements, except the first one. In Table 4 the results of the ANOVA test can be found, and in Additional file 1 the post-hoc Bonferroni tests indicate for each element which practice types differs significantly from each other. Considering the final score, this should be interpreted as reasonably good support for CIC for the capitation practices and basic support for CIC for both types of the fee-for-service practices.

**Table 4:** Average ACIC scores by type of GP practice

ACIC elements	Fee-for-service				Capitation		
	Mono-disciplinary		Multi-disciplinary		Multi-disciplinary		
	x̄	sd	x̄	sd	x̄	sd	*p*-value*
1. Health Organisation	6.22	1.48	5.77	1.37	6.59	1.75	0.28
2. Community linkages	2.53	1.41	3.35	1.65	5.08	1.13	< 0.001
3.a Self-management support	3.19	1.51	4.82	0.97	6.63	0.92	< 0.001
3.b Decision support	3.06	1.09	3.84	0.96	4.72	0.75	< 0.001
3.c Delivery system design	2.90	1.80	3.99	1.75	6.28	1.25	< 0.001
3.d Clinical information system	2.27	1.22	4.48	1.14	7.16	0.99	< 0.001
**Total score**	**3.36**	**1.02**	**4.38**	**0.83**	**6.08**	**0.77**	** < 0.001**

^*^ANOVA test; *sd* standard deviation

In Table 5, the results of the regression analyses are presented, there were three models calculated, always adding extra possible influencing factors. In model 1, we see that the financing system is significantly related to the ACIC score. After taking the other practice characteristics into account (in model 2 and 3), GP practices within the capitation system had a significantly higher overall ACIC score compared to GP practices within the fee-for-service system. The strength of this relation, however, decreases in particular when controlling for whether the practice had a nurse (model 2), indicating a mediating effect in addition to the direct effect of the financing system. Bivariate statistics showed that GP practices within the capitation system more often have a nurse, and in model 2, we observed that GP practices with a nurse scored significantly higher on the ACIC. The total ACIC score was not related to the inclusion of a dietician in the practice (model 2). A total of 14% of the variance in ACIC scores between the practices can be ascribed to the presence or absence of these paramedics.

**Table 5:** Multivariate regression analysis on the total ACIC score. Multivariate regression analysis relating (1) the financing system and region (Model 1), (2) the availability of nurses and diaticiens (Model 2) and (3) the availability of a secretary (Model 3) to the total ACIC score

	Model 1			Model 2			Model 3		
	b	se		b	se	sign	b	se	sign
**Financing system (ref. ‘fee-for-service’)**									
Capitation	**2.392**	0.269	< 0.001	**1.130**	0.313	< 0.001	**1.073**	0.299	< 0.001
**Region (ref. ‘Antwerp’)**									
Ghent	0.779	0.264	0.004	0.946	0.218	< 0.001	1.092	0.216	< 0.001
Campine	0.973	0.291	0.001	0.961	0.237	< 0.001	0.936	0.226	< 0.001
**Nurse (ref. ‘No’)**				**1.423**	0.275	< 0.001	**1.149**	0.282	< 0.001
**Dietician (ref. ‘No’)**				0.279	0.203	0.175	0.129	0.202	0.526
**Secretary (ref. ‘No’)**							**0.650**	0.247	0.011
Adjusted R^2^	0.588			0.726			0.751		

*se* standard error

In Model 3, we see that GP practices with a secretary also scored significantly higher on the ACIC, irrespective of the financing type of the practice and whether or not there was a dietician and/or a nurse. By adding ‘secretary’ to the model, an additional 3% of the variance was explained. Finally, 75% of the variance in the ACIC scores was explained by the financing system, region, and having a nurse, dietician or secretary. When looking at the last model, nurses have the highest impact, followed by being a capitation practice and having a secretary. This last model was re-estimated for the separate elements of the ACIC (see Additional file 2).

### Organisation of the HC delivery system

The region of Ghent scored higher than the region of the Campine, which scored higher than the region of Antwerp. As these scores were based on the interview with one single respondent for each region — a meso-level stakeholder — no statistical analysis could be performed. The reason the region of Ghent scored higher than the other regions is mainly due to the fact that they have set up a health council since a decade. This council devised an improvement strategy, with measurable goals, which are reviewed routinely. In the other regions, the improvement strategy was rather implicit. Additionally, senior leaders visibly participate in the efforts for the improvement of chronic care in Ghent. The region of the Campine scored higher than Antwerp because they have set up a prevention centre, in which multiple behaviour-change interventions are available. The revenues from the GPs are used to fund the centre. In all the regions, scores for incentives and regulations for chronic illness care were low. Respondents mentioned that the current Belgian financing system does not provide incentives to improve quality of care, and that they cannot influence this. One respondent mentioned that in their region, they try to stimulate professional satisfaction instead; to stimulate the quality of chronic care in a different way.

### Community linkages

Having a nurse and being a capitation practice had a significant impact on element 2 of the CCM: community linkages. Practices with nurses more often referred patients with T2D to initiatives organised by other partners in the community. These could be exercise classes, cooking classes, peer-support programmes, exercise coaching or social care. In practices where nurses were given extensive responsibility and also actively sought to change patient behaviour, referring patients to other initiatives was often their task and they sometimes actually had a list of places to which they could refer patients with T2D. In practices where the role of the nurse was mainly technical, they more often considered this to be the task of the GPs. In most capitation practices, the tasks of the nurses were elaborated. While in some fee-for-service practices that was also the case; however, in others, they mainly performed technical tasks. Most GPs, with few exceptions, often did not have the time or were not aware of community initiatives. Real collaboration with these initiatives was rare, and when it did occur, it was in some of the capitation centres that had dedicated staff for this purpose — a health promoter.

### Self-management support

Being a capitation practice, having a nurse, dietician and secretary impacted the score for self-management support, with the nurse having the biggest impact. Nurses often adopt a more structured approach than GPs and, on the basis of a protocol, discuss all aspects that can be affected by T2D. Some participants also indicated that patients found it easier to tell their concerns to the nurse than to the GP. Explaining to patients how they should check their blood pressure or glucose level themselves was often part of the nurse’s job. Not all nurses were responsible for effective interventions related to behavioural change; especially when their tasks were purely technical, they left this to other healthcare providers. Dieticians are eminently in charge of changing behaviour, but in contrast to the nurse, they mostly served as an optional provider; not all patients went to see the dietician. Some dieticians even said that they received very few referrals from the GP and mainly provided care to patients visiting them independently. Secretaries helped in arranging appointments with educators and other providers and in such a way, supported self-management.

### Decision support

Capitation practices scored higher on decision support compared to multidisciplinary fee-for-service practices, which scored higher than monodisciplinary practices. However, the difference between the practices in the decision support element was the smallest of all elements. In the regression analysis, the different parameters were all borderline significant and only explained 40% of the differences between practices. It was observed that using guidelines, involving specialists or taking education also happens in monodisciplinary practices. However, multidisciplinary practices made more use of guidelines, and in capitation practices, it was sometimes observed that guidelines were adapted into a specific practice protocol to cooperate across disciplines. Many nurses and dieticians follow regular training on diabetes and a few nurses had followed the general practice nurse training, where the organisation of chronic conditions is discussed in detail. Finally, informing patients about guidelines is also a task that nurses and dieticians take on—they use leaflets or other material for education more often than doctors.

### Delivery system design

Concerning the delivery system design element, compared to all other elements, the parameters in the regression analysis were able to explain most (81%) of the differences between the practices. Being a capitation practice, having a nurse and having a secretary had a large impact, whereas having a dietician did not have an impact. When a GP practice decides to work with a nurse or secretary, tasks can be better divided between them. Practices with the highest scores hold meetings regularly and also have prior agreements on which care provider will take on which tasks. When the group of caregivers grows larger, cooperation runs more smoothly if one caregiver takes the lead in organising the care for the patients with T2D. This dialogue between healthcare providers is one of the points where practices with nurses differ from practices with only a dietician as the paramedic. The dietician is often more external to the group, where, in the fee-for-service practices, the dietician rents a consultation room from the doctors as an independent provider and is, therefore, less accountable to them, in contrary to the nurse and secretary, who are paid by the GP budget. Dieticians also often spend fewer hours in the GP practice and can combine it with consultations in other places. Practices with a nurse often have more concrete agreements about when the patient should come for a consultation, usually every three months. Nurses and secretaries are also more likely to actively contact or call the patient if they do not show up and assure appointments in the hospital are planned, if needed. Planned consultations, in which only the chronic disease is managed and who are prepared by certain activities, such as a blood test, are more common in practices with nurses. Different models are possible, such as a consultation with the nurse and shortly afterwards with the doctor, or a blood collection beforehand via the lab. The nurses used their protocol to plan the content of their consultations; in some practices, this was even arranged periodically, with a different element of follow-up each year. Nurses also often had more time per consultation than doctors. Most importantly, when patients visit their doctors, they often bring other complaints (apart from the chronic disease), while only asking for a prescription for their chronic medication at the end of the consult. Obviously, in such a way it is difficult to manage diseases such as T2D, respondents said.

### Clinical information system

Being a capitation practice and having a nurse had a significant effect on the score on the clinical information system dimension. We observed that the electronic medical record is often better developed in practices with a nurse. This is often an essential tool for communication between healthcare providers within a practice. Practices with nurses are more likely to be able to extract a list of the diabetes patients from the system and work more often with schedules and reminders. Developing and recording a clear care plan with goals, both clinical and self-management, was more common in practices with a nurse, with some practices having developed their own template for this. Although, there was also a big difference between practices where the nurse merely performed technical tasks and practices in which nurses did take up more advanced tasks, the latter being more common in capitation practices. Also, there was a notable difference with practices that only had a dietician: in those practices, the medical records were usually not shared, but referral letters and reports were used. Lastly, in the capitation practices, specific medical software was used, which has the function of planning and reminders in a prominent place, which was used by all the capitation practices.

## Discussion

### Main findings

This study is the first to describe the implementation of IC for T2D patients in regular care, over a whole territory. The scores of the fee-for-service practices in Belgium correspond to basic support for chronic illness care, while the scores for the capitation practices indicated reasonably good support. We were able to find the most important influencing factors, as 75% of the variance in the ACIC scores was explained by the financing system, region and having a nurse, dietician or secretary.

A first important finding is the fact that the presence of a nurse had the highest impact on the values that mirror the implementation of IC. However, an important spread between the approaches of different nurses was observed. Whereas some still mainly focussed on technical tasks, others were nearly completely in charge of disease management. Previous studies have proven the positive impact of nurse-led models on T2D care [38, 39] and also the impact on the patients’ HbA1c levels [40, 41]. Self-developed protocols [38], patient empowerment and emotional support [42], quality of communication [43] and managing care through care plans [44] have been suggested as mechanisms for better care by nurses. In our research, additional mechanisms have been identified: nurses enhance IC due to their focus on chronic care, structured approach, teamwork and their linkages with other HCWs. Belgian GPs are willing to integrate nurses into their practice [45], but face a lack of governmental support and funding.

A second observation is that a secretary also had a significant effect on the implementation of integrated care. The role of a secretary or receptionist is rarely studied and therefore we did not predict this outcome. Some researchers studied their role in the continuity of care, by means of providing repeat prescriptions [46], communicating test results [47] and managing the waiting room [48]; however, their role is still debated [49, 50]. Our study is the first to validate the role of a secretary in chronic care quantitatively.

A third finding is the added value of working with multiple disciplines under one roof. In this regard, it is interesting to analyse developments in T2D care in Belgium. Diabetes care has a special status in Belgium, as it is one of the two diseases for which a care trajectory exists in primary care. This trajectory was based on a pilot study, in which the ACIC was also used to measure care delivery. At the time, all practices in that region were monodisciplinary fee-for-service practices. A significant increase in the ACIC scores from 1.45 before the project to 5.5 after the project was observed [28]. It is noteworthy that in our study, both the monodisciplinary and multidisciplinary fee-for-service practices scored in between these two scores, and only the capitation practices scored higher, despite the efforts of the government to scale up the project and other reforms intending to implement IC in the decade after. Belgian researchers warned before that the care trajectory, in which diabetes education was foreseen by an external educator in primary care, could fail because both patients and physicians would face barriers to visiting an extra external provider [51, 52], but also that there were limits to the clinician-centred model, as some of them did not want to cooperate [53], or that it became too complex to coordinate [54]. Indeed, another study [55] revealed that only 16% of the eligible patients participated in the trajectory. Bringing services as close to the patients as possible is crucial for participation, and for many patients, their GP practice is what they see as their ‘medical home’ [56]. Additionally, for practitioners as well, real multidisciplinary teamwork is only possible if they work under the same roof and is key to reaching healthcare quality improvement [14].

A fourth and last observation is the importance of the financing system. Capitation practices especially developed a model in which task-shifting towards nurses was more extensive. However, due to the observational methodology, no causal relationship can be made. The financing system could be a cause of the differences, but an alternative explanation could be that the longer a nurse works in a practice, the more responsibilities she gets. This is probable as it was noticed that in the capitation practices, teamwork was already a tradition for a long period, whereas most multidisciplinary fee-for-service practices only recently employed nurses. Another alternative explanation is that providers in the capitation system have more experience with diabetes and therefore score higher. This is probable, as the preliminary analysis from our follow-up study [57], indicates that capitation practices have a prevalence of 12% of patients with diabetes, compared to 8% in the other practices. On the other hand, practitioners in fee-for-service practices also complained that their financing system withholds them from delegating more tasks, as the more tasks are delegated, the less they earn. Whereas in the capitation practices, there was complete freedom to organise and divide tasks as suited the practice best. Practitioners working in this system also report a better work-life-balance and a lower workload, which may be a reason they have more time for chronic care [58]. Previous studies have signalled the dominant fee-for-service system as a barrier to IC in Belgium [54, 59] and the superiority of the capitation system concerning the quality of care in the field of diabetes care [60]. Even though the capitation system scored significantly better in our study, it is debated whether it is the ideal system to stimulate qualitative chronic care, as it could provide caregivers with the option to enrol more patients than can be taken care of [61]. Other types of payment such as bundled payments [62], global payment and pay-for-performance [61] have been suggested. Our research acknowledges the importance of integrated financing, as the dieticians in our sample (paid independently) were much less integrated compared to the nurses (paid from the same budget as the GPs’). The discussion should therefore go beyond the traditional fee-for-service versus capitation opposition, as there are many more dimensions that define the integration of payment systems [63], such as provider coverage, as we envisage.

### Strengths and limitations

There are some limitations to our study. First, as this was an observational study, no causal interpretations could be made. While it could be possible that adding a nurse or secretary to a GP practice raises the quality of care, it could also be the case that for instance, GPs who already strive for high quality of care are more keen on taking a nurse or secretary. Next, due to the COVID-19 pandemic, we had to change the methodology slightly; however, there were no considerable differences between the practitioners interviewed live or by video call. By the time the methodology had to be changed, the researchers were experienced in collecting the data and assessed that all needed information could be collected through video calls. Then, an important amount of practitioners refused to participate, which could have led to some bias as the ones willing to participate could also have scored higher. Moreover, the refusal rate was highest in the lowest scoring group, therefore, if any distortion of the effect is present, it will probably be an underestimation of the difference between the practice types. Lastly, as secretaries were not interviewed, the information about their behaviour is indirect and limited. Further research on their role within chronic care is recommended.

The observational design of the study is also a strength. Whereas the ACIC is often used in study settings containing an intervention, which already select practices or regions wanting to participate in the intervention, our participating practices did not have to commit to anything. Therefore, our results are more unbiased and represent the implementation of the scale-up of IC in the real world, irrespective of any intervention. Other strengths of our study are to be found within the methodology. Firstly, two researchers filled the ACIC score independently. It is a unique way of using the questionnaire and increases the reliability. Next, using both quantitative and qualitative methods allowed us not only to discover which practices scored higher, but also to explore some reasons for this variation. Lastly, practices in three urban and rural regions in Flanders were sampled, hence a good spread was reached.

## Conclusions

Our study confirms the importance of the role of a nurse in care for chronic diseases within primary care and adds to that the relevance of the secretary and the integration of the payment system. Belgian policymakers are encouraged to rethink the role of paramedics in primary care. Currently, there exists some financial support for a secretary. In a recent reform, there was decided that the budget for this support could also be used to attract a practice nurse. The budget is however limited and cannot cover the whole wage, but more importantly, it was not raised, so practices have to choose between a secretary and a practice nurse, whereas both are proven to be indispensable. The question should be raised whether such marginal policy changes will change how practices organise themselves. Ideally, policymakers take the whole system into account, and rethink the payment system, making it more integrated, not only by changing the balance from fee-for-service towards more capitation payment but also by considering integrating payment of primary care teams.

When looking at the scale-up of the IC we demonstrated that the variability between different practices is major. Real multidisciplinary teamwork is facilitated hugely when working under the same roof. As we found evidence for the link between the structure and the processes within one organisation it is also important for other countries to consider different types of organisations in primary care practices when wanting to scale-up IC for chronic diseases. Uniform roll-out across a system containing multiple types of practices may not be successful, it could be more fruitful to identify the optimal model and invest in that one.

## Supplementary Information

**Additional file 1.**

**Additional file 2.**

## Data Availability

The datasets used and/or analysed during the current study are available from the corresponding author on reasonable request.
