# Supplementary material for: Scale-up of a chronic care model-based programme for type 2 diabetes in Belgium: a mixed-methods study
Source: BMC Health Serv Res. 2023 Feb 9;23:141. doi: 10.1186/s12913-023-09115-1 (PMC9911183; doi:10.1186/s12913-023-09115-1)
Supplement: Supplementary file 1 — Additional file 1. [file 12913_2023_9115_MOESM1_ESM.docx]

Additional table 1

| Multiple Comparisons | | | | | |  |
| --- | --- | --- | --- | --- | --- | --- |
|  | | | | | |  |
| Bonferroni | | | | | |  |
| Dependent Variable | | | Mean Difference | Std. Error | Sig. |  |
|  |  |  |  |  |  |  |
| 1. Organisation of the healthcare delivery system | monodisciplinary | multidisciplinary, fee-for-service | 0.45 | 0.45 | 0.952 |  |
|  |  | multidisciplinary, capitation | -0.37 | 0.46 | 1.000 |  |
|  | multidisciplinary, fee-for-service | monodisciplinary | -0.45 | 0.45 | 0.952 |  |
|  |  | multidisciplinary, capitation | -0.82 | 0.51 | 0.341 |  |
|  | multidisciplinary, capitation | monodisciplinary | 0.37 | 0.46 | 1.000 |  |
|  |  | multidisciplinary, fee-for-service | 0.82 | 0.51 | 0.341 |  |
| 2. Community linkages | monodisciplinary | multidisciplinary, fee-for-service | -0.82 | 0.42 | 0.163 |  |
|  |  | multidisciplinary, capitation | -3 | 0.43 | **0.000** |  |
|  | multidisciplinary, fee-for-service | monodisciplinary | 0.82 | 0.42 | 0.163 |  |
|  |  | multidisciplinary, capitation | -1.73 | 0.47 | **0.002** |  |
|  | multidisciplinary, capitation | monodisciplinary | 2.55 | 0.43 | **0.000** |  |
|  |  | multidisciplinary, fee-for-service | 1.73 | 0.47 | **0.002** |  |
| 3.a Self-management support | monodisciplinary | multidisciplinary, fee-for-service | -1.62 | 0.36 | **0.000** |  |
|  |  | multidisciplinary, capitation | -3.44 | 0.38 | **0.000** |  |
|  | multidisciplinary, fee-for-service | monodisciplinary | 1.62 | 0.36 | **0.000** |  |
|  |  | multidisciplinary, capitation | -1.82 | 0.41 | **0.000** |  |
|  | multidisciplinary, capitation | monodisciplinary | 3.44 | 0.38 | **0.000** |  |
|  |  | multidisciplinary, fee-for-service | 1.82 | 0.41 | **0.000** |  |
| 3.b Decision support | monodisciplinary | multidisciplinary, fee-for-service | -0.78 | 0.29 | **0.025** |  |
|  |  | multidisciplinary, capitation | -1.66 | 0.30 | **0.000** |  |
|  | multidisciplinary, fee-for-service | monodisciplinary | 0.78 | 0.29 | **0.025** |  |
|  |  | multidisciplinary, capitation | -0.88 | 0.33 | **0.028** |  |
|  | multidisciplinary, capitation | monodisciplinary | 1.66 | 0.30 | **0.000** |  |
|  |  | multidisciplinary, fee-for-service | 0.88 | 0.33 | **0.028** |  |
| 3.c Delivery system design | monodisciplinary | multidisciplinary, fee-for-service | -2.21 | 0.33 | **0.000** |  |
|  |  | multidisciplinary, capitation | -4.88 | 0.35 | **0.000** |  |
|  | multidisciplinary, fee-for-service | monodisciplinary | 2.21 | 0.33 | **0.000** |  |
|  |  | multidisciplinary, capitation | -2.67 | 0.38 | **0.000** |  |
|  | multidisciplinary, capitation | monodisciplinary | 4.88 | 0.35 | **0.000** |  |
|  |  | multidisciplinary, fee-for-service | 2.67 | 0.38 | **0.000** |  |
| 3.d Clinical information system | monodisciplinary | multidisciplinary, fee-for-service | -1.09 | 0.49 | 0.087 |  |
|  |  | multidisciplinary, capitation | -3.38 | 0.50 | **0.000** |  |
|  | multidisciplinary, fee-for-service | monodisciplinary | 1.09 | 0.49 | 0.087 |  |
|  |  | multidisciplinary, capitation | -2.29 | 0.55 | **0.000** |  |
|  | multidisciplinary, capitation | monodisciplinary | 3 | 0.50 | **0.000** |  |
|  |  | multidisciplinary, fee-for-service | 2.29 | 0.55 | **0.000** |  |
| Total score | monodisciplinary | multidisciplinary, fee-for-service | -1.01 | 0.27 | **0.001** |  |
|  |  | multidisciplinary, capitation | -2.71 | 0.28 | **0.000** |  |
|  | multidisciplinary, fee-for-service | monodisciplinary | 1.01 | 0.27 | **0.001** |  |
|  |  | multidisciplinary, capitation | -1.70 | 0.30 | **0.000** |  |
|  | multidisciplinary, capitation | monodisciplinary | 2.71 | 0.28 | **0.000** |  |
|  |  | multidisciplinary, fee-for-service | 1.70 | 0.30 | **0.000** |  |
