# Supplementary material for: Scale-up of a chronic care model-based programme for type 2 diabetes in Belgium: a mixed-methods study
Source: BMC Health Serv Res. 2023 Feb 9;23:141. doi: 10.1186/s12913-023-09115-1 (PMC9911183; doi:10.1186/s12913-023-09115-1)
Supplement: Supplementary file 2 — Additional file 2. [file 12913_2023_9115_MOESM2_ESM.docx]

Multivariate regression analysis on the different ACIC elements, model 3
Financing system, region, and organizational factors regressed on the different elements of the ACIC

|  | ACIC 2 | | | ACIC 3.a | | | ACIC 3.b | | | ACIC 3.c | | | ACIC 3.d | | |
| --- | --- | --- | --- | --- | --- | --- | --- | --- | --- | --- | --- | --- | --- | --- | --- |
|  | b | se | sign | b | se | sign | b | se | sign | b | se | sign | b | se | sign |
| **Financing system** (ref. ‘fee-for-service’) |  |  |  |  |  |  |  |  |  |  |  |  |  |  |  |
| Capitation | **1.325** | 0.445 | 0.004 | **1.158** | 0.485 | 0.02 | 0.779 | 0.389 | 0.05 | **1.716** | 0.419 | <,001 | **1.459** | 0.634 | 0.025 |
| **Region** (ref. ‘Antwerp’) |  |  |  |  |  |  |  |  |  |  |  |  |  |  |  |
| Ghent | **1.268** | 0.321 | <0.001 | **1.031** | 0.35 | 0.005 | 0.085 | 0.281 | 0.763 | -0.008 | 0.302 | 0.979 | 0.675 | 0.457 | 0.145 |
| Campine | **2.227** | 0.337 | <0.001 | 0.521 | 0.367 | 0.161 | **0.66** | 0.294 | 0.029 | -0.11 | 0.317 | 0.73 | **1.151** | 0.48 | 0.02 |
| **Nurse** (ref. ‘No’) | **1.264** | 0.42 | 0.004 | **1.221** | 0.457 | 0.01 | 0.673 | 0.367 | 0.071 | **2.104** | 0.395 | <0.001 | **1.634** | 0.598 | 0.008 |
| **Dietician** (ref. ‘No’) | 0.03 | 0.301 | 0.921 | **0.673** | 0.328 | 0.044 | -0.225 | 0.263 | 0.397 | 0.45 | 0.284 | 0.118 | -0.155 | 0.429 | 0.72 |
| **Secretary** (ref. ‘No’) | 0.41 | 0.367 | 0.269 | **0.852** | 0.4 | 0.037 | 0.624 | 0.321 | 0.057 | **1.103** | 0.346 | 0.002 | 0.913 | 0.523 | 0.086 |
| Adjusted R^2^ | 0.632 |  |  | 0.619 |  |  | 0.387 |  |  | 0.812 |  |  | 0.505 |  |  |

se: standard error; We estimated the same model (model 3 of Table 5: with financing system, region, and the availability of a nurse, dietician, and secretary in the primary care practice as independent variables) on the 5 different ACIC elements (ACIC 2, 3.a, 3.b, 3.c and 3.d as the dependent variables).
